# VAPA, an Innovative “Virus-Acquisition Phenotyping Assay” Opens New Horizons in Research into the Vector-Transmission of Plant Viruses

**DOI:** 10.1371/journal.pone.0023241

**Published:** 2011-08-10

**Authors:** Alexandre Martinière, Jean-Luc Macia, Guillaume Bagnolini, Chiraz Jridi, Aurélie Bak, Stéphane Blanc, Martin Drucker

**Affiliations:** Equipe CaGeTE, INRA, UMR BGPI, Campus International de Baillarguet, Montpellier, France; University of Wisconsin-Milwaukee, United States of America

## Abstract

Host-to-host transmission—a key step in plant virus infection cycles—is ensured predominantly by vectors, especially aphids and related insects. A deeper understanding of the mechanisms of virus acquisition, which is critical to vector-transmission, might help to design future virus control strategies, because any newly discovered molecular or cellular process is a potential target for hampering viral spread within host populations. With this aim in mind, an aphid membrane-feeding assay was developed where aphids transmitted two non-circulative viruses [cauliflower mosaic virus (CaMV) and turnip mosaic virus] from infected protoplasts. In this assay, virus acquisition occurs exclusively from living cells. Most interestingly, we also show that CaMV is less efficiently transmitted by aphids in the presence of oryzalin—a microtubule-depolymerising drug. The example presented here demonstrates that our technically simple “virus-acquisition phenotyping assay” (VAPA) provides a first opportunity to implement correlative studies relating the physiological state of infected plant cells to vector-transmission efficiency.

## Introduction

Transmission is a critical step in the infection cycle of every virus, because it controls dispersal in space and time, thus directly influencing epidemiology. Understanding this process is, besides being of genuine scientific interest, crucial to the development of alternative disease control strategies. Many viruses, especially plant viruses, are vector-transmitted by insects. Among insect vectors, aphids play a dominant role as they transmit about one-third of all known plant viruses (reviewed in [1]). This is due partly to their non-destructive feeding behaviour. When alighting on a new plant, aphids first insert their stylets (the proboscis-like mouth parts) into epidermal and mesophyll cells in order to test plant palatability. These test punctures last only seconds and usually preserve plant cell integrity. Only when the plant is “approved” by the aphid do more test punctures guide the stylets to the phloem, where aphids settle for prolonged feeding from the sieve tube sap. When the plant is not a host for the aphid, it soon departs, after very few test punctures, and continues the search for a suitable host (reviewed in [2]). Aphids can acquire viruses efficiently during one of these feeding steps, or even during both steps, depending on the viral species (e.g. [3]).

Vector-transmission of plant viruses can be classified into two major categories: circulative and non-circulative transmission. In circulative transmission, the acquired virus circulates from the intestine through the vector body to the salivary glands, and is then inoculated with the saliva into a new host. At least equally important is the non-circulative transmission that is used by about half of all known plant viruses (reviewed in [4]). In this transmission mode, transmissible virus particles are never internalised within the vector body; the association is exclusively external, and viruses attach to the chitin cuticle lining the food and/or salivary canals within the stylets bundle during ingestion of sap or infected cell content. The inoculation into another host plant is believed to occur upon release of the virus particle from the attachment sites, most probably by the action of saliva [5], [6]. For the non-circulative cauliflower mosaic virus (CaMV), the attachment sites have been shown to be located exclusively at the extreme tip of the stylets bundle, within the so-called common duct where the food and salivary canals combine. In fact, the attachment site of CaMV is a proteinaceous receptor(s) localised to a specific morphological structure called the acrostyle [7], [8]. Because other non-circulative viruses are also retained within the common duct [5], [6], it is likely that they also use the acrostyle for transmission, although direct experimental proof is lacking.

Non-circulative transmission has been regarded historically as a non-specific event where vectors acquire viruses “by chance” during feeding and drag them along to a new host in their contaminated stylets. However, in recent decades, evidence is accumulating that non-circulative transmission of plant viruses is a specific phenomenon, and increasing layers of sophistication are still being unravelled. There is clearly virus-vector specificity [9], [10]; many viruses encode so-called “helper proteins”—molecular bridges linking virus particles to the stylet cuticle that are mandatory for transmission (reviewed in [11])—and CaMV induces the formation in infected cells of a viral inclusion body that is specialised for the control of vector-transmission [12], [13]. Most surprisingly, a recent structural study of the CaMV “transmission body” (TB) suggested that physiological conditions within the infected cell can affect TB stability, and consequently transmission efficiency [14]. The TB of CaMV is thus helping to reveal a fascinating new level of complexity of transmission. In fact, many aspects of the interaction between viruses and host plant cells, other than those involved in viral replication, accumulation and cell-to-cell movement, participate in the success of vector transmission. Exploring this new horizon will be difficult unless an amenable tool is developed that would allow correlative studies between the physiological state of the host plant cell and the success of vector transmission.

In this technical paper, we describe an *in vitro* acquisition system using infected protoplasts that allows us to control and manipulate the physiological state of the cells precisely, for example through the use of bio-active compounds and/or drugs, and at the same time to examine virus acquisition and transmission efficiency. Because we were able to apply this technique successfully to both caulimoviruses and potyviruses, it is likely also transferable to other non-circulative (and perhaps also to circulative) viral species.

## Methods

### Aphid maintenance

Aphids (*Myzus persicae* Sulz.) were maintained on eggplant in insect-proof cages with a photoperiod of 14 h day at 23°C, and 10 h night at 18°C. Colonies were transferred to new plants once a week.

### Purification of infected protoplasts

Protoplasts were purified from infected leaves essentially as described [13]. Briefly, 14-day-old turnip plants (*Brassica rapa* cv. ‘Just Right’), maintained in a greenhouse under controlled long-day conditions (16 h day at 25°C, 8 h night at 19°C), were rub-inoculated with CaMV (Cabb B-JI strain, [15]) or TuMV (UK1 isolate [16]) on first true leaves numbers 1 and 2. Two weeks post-inoculation, systemically infected leaves were soaked for 3 min in 0.5% Domestos (Unilever), washed 3 times with tap water, and once with MilliQ water (Millipore). They were then incubated overnight with 0.5% cellulase R10 and 0.05% macerozyme (both from Yakult) in protoplast buffer (0.5 M mannitol, 1 mM CaCl_2_, 10 mM MES, pH 5.5) in the dark at 25°C. The next day, protoplasts were liberated from the leaves by gentle shaking and, after incubation for another 30 min, filtered through 1 layer of Miracloth (Merck). Protoplasts were washed 3 times with protoplast buffer by centrifugation (80 g for 5 min in a swing-out rotor). Protoplasts were resuspended in protoplast buffer, split into aliquots as required, and gently shaken (4 r.p.m.) in Eppendorf tubes for 1–2 h at room temperature to allow recovery from centrifugation.

### Aphid acquisition assays

**Figure 1:**
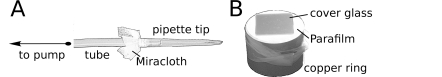
Experimental set-up of a typical VAPA (virus-acquisition phenotyping assay) transmission experiment. **A.** To construct the aphid harvesting device, a 1000 µl pipette tip with the utmost 2–3 mm cut off is attached to a silicon tube, with a Miracloth net squeezed in between. The tube is connected to a vacuum source (mechanical pump or human respiratory system) and aphids are sucked up into the pipette tip by negative pressure and retained by the net. **B.** The virus-acquisition phenotyping apparatus consists of a copper ring sealed with a Parafilm M membrane. Aphids placed in the ring are attracted to the membrane by a light source (not shown); protoplasts are then deposited onto the membrane and spread evenly with a cover glass. After a defined acquisition access period, aphids are transferred with an artist's paint-brush to test plants for inoculation.

Apterous adult aphids were harvested with the help of an electric chemical duty vacuum/pressure pump (model WP 6122 050, Millipore). One end of a silicon vacuum tube was connected to the vacuum outlet of the pump. The other end was covered with a small piece of Miracloth (Merck), and a P1000 pipette tip with the end (2–3 mm) of the tip cut off, was squeezed onto the tube. The ensemble served as an aphid retention reservoir, with the Miracloth net acting as an aphid stop filter (Figure 1A). To increase harvesting capacity, sometimes two to three vacuum tubes were connected to the pump. Aphids were gently aspirated (−15 to −20 kPa pressure) with this equipment. If no mechanical pump is to hand, virtually identical results are obtained with the tubing connected to a mouth, with the aphids being aspirated by human respiratory power; however, care should be taken to avoid hyperventilation. Whatever the harvesting method, when an adequate number of aphids was collected, they were carefully placed in copper rings (3.2 cm diameter, 3 cm height), sealed with a stretched Parafilm M membrane (Pechiney Plastic Packaging) on the lower side. The copper rings were then inverted, placed on black tiles in a humid chamber with the membrane side up, and a light source (desktop lamp) was placed above the humid chamber to attract the aphids to the membrane. After a defined time of fasting, 200 µl of protoplast suspension was deposited on the upper side of the membrane and covered with a cover glass (20*20 mm; Figure 1B). Aphids were allowed the allocated time for virus acquisition, and then placed, using a soft hog bristle brush (size 6, model 831, Raphaël Child Fun Line), in groups of 10 aphids per plant on test plants for inoculation. Only aphids that were in contact with the feeding solution (i.e. those beneath the cover glass) were transferred to test plants. Test plants were 1-week old turnip seedlings that were still at the cotyledon stage. Batches of 24 plants in a plant cultivation tray were inoculated using aphids from one copper tube, and 12 batches (thus 12 copper tubes) were inoculated per experiment. The inoculation time allowed was 1 hour, after which aphids were killed by spraying the plants with the aphid-specific insecticide Pirimor G (Certis). Three weeks later, transmission rates were determined by visual inspection of the test plants: only those plants showing unambiguous mosaic symptoms on the leaves were considered as infected, all others as healthy.

For plant-to-plant transmission experiments, aphids were allowed a 15-min acquisition access period on detached infected leaves. Then aphids were transferred to test plants at a ratio of 2 aphids per plant. To test directly the effect of oryzalin on aphids, in vitro acquisition assays using purified CaMV particles and recombinant P2 and P3 were carried out as described [17] in the presence of 50 µM oryzalin or its solvent DMSO.

### Incubation and protoplast treatments

CaMV-infected protoplasts were disrupted by gentle ultrasonication (five 5 sec strokes with a Bioblock Vibra Cell 72434 ultrasonicator operated at 60% power). TuMV-infected protoplasts were disrupted by passing the protoplasts 5 times through a 0.45 mm-diameter syringe needle. Disruption was verified by wide field microscopy.

Protoplasts were treated with oryzalin (Supelco) for 60 min by adding the drug to 10–50 µM final concentration from a 1 mM stock solution in DMSO. Control protoplasts for this experiment were mock-incubated with an identical volume of DMSO.

### Statistics

A non-parametric, several independent samples Krustal-Wallis test was used to test the effect of duration of acquisition, and pre-acquisition fasting on transmission rates of CaMV and TuMV. The effect on virus transmission rate of oryzalin and protoplast disruption by shearing and ultrasound was tested separately using a non-parametric two independent samples Mann-Whitney test. All statistical analyses were carried out using the SPSS software package for windows version 17.0 (SPSS Inc., Chicago, IL, USA). *P*-values<0.05 were considered statistically significant.

## Results

**Figure 2:**
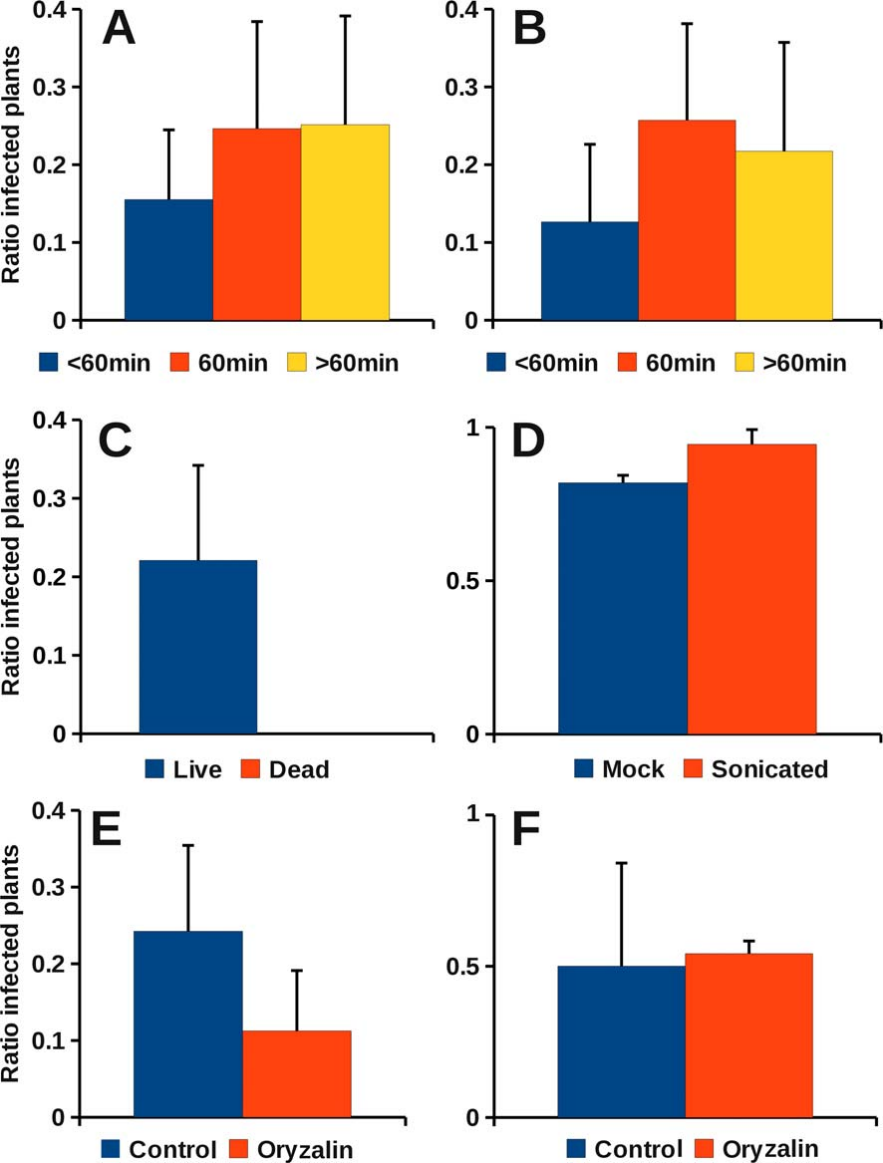
Transmission of CaMV from infected protoplasts. **A.** Influence of acquisition time on transmission of CaMV from infected protoplasts. After a fasting period of 60 min, aphids were allowed access to protoplasts for 15–45 min (<60 min), 60 min, or 75–180 min (>60 min) before transfer to healthy test plants. Acquisition times shorter than 60 min diminished somewhat, but not significantly transmission (*P* = 0.142; df = 2; Kruskal-Wallis test). Also acquisition times longer than 60 min were not associated with any significant difference in transmission rate compared to a 60-min acquisition. The graph presents data from 57 assays of 24 test plants each (9 lots for acquisition times shorter than 60 min, 17 lots for an acquisition time of 60 min, and 31 lots for acquisition times longer than 60 min). **B.** Effect of fasting on CaMV transmission from infected protoplasts. Aphids were starved for between 15 and 45 min (<60 min), for 60 min, or between 90 and 240 min (>60 min). They were then allowed a 60-min acquisition access period on protoplasts before transfer to test plants for inoculation. Under our conditions, no prominent effect of starvation on transmission was observed, although starving times shorter than 60 min seemed to reduce transmission. However, the effect was not significant (*P* = 0.083; df = 2; Kruskal-Wallis test). Data are from 59 tests using 24 plants each (9 assays for fasting of less then 60 min, 19 assays for a fasting time of 60 min, and 31 assays with fasting times longer then 60 min. Note that the data for 60 min fasting contains data from (A). **C.** Transmission of CaMV from protoplasts absolutely requires living cells. Intact (live) or ultrasonicated (dead) infected protoplasts were offered to aphids in acquisition assays. Only living protoplasts supported transmission. Data are from 11 assays of 24 test plants for each condition. The difference between the two acquisition conditions was highly significant (*P*<0.001; Mann-Whitney test). **D.** Ultrasonication does not inactivate CaMV. Infected protoplasts were disrupted by ultrasonication and then transmitted by rub-inoculation. The graph shows that ultrasonicated protoplasts performed at least as well as intact protoplasts in mechanical transmission. Three lots of 24 plants were tested per condition. **E.** Oryzalin inhibits transmission of CaMV. Protoplasts were incubated for 60 min with DMSO (Ctl) or 10–50 µM oryzalin (Ory) before being offered to aphids for virus acquisition. After a 60-min access period, aphids were transferred to test plants for inoculation. The data shown are from 11 sets of 24 plants for each condition. One set was excluded from analysis because it was an outlier. The difference between the two conditions is significant (*P* = 0.014; Mann-Whitney test). **F.** Oryzalin does not impair in vitro acquisition of CaMV. Purified CaMV particles were mixed with helper component P2 and P3 and offered with or without 50 µM oryzalin to aphids for a 30-min acquisition access feeding period, before the insects were used for inoculation of test plants (10 aphids per plant). Three 24 plant lots were used per condition. All graphs present mean values ± standard deviation.

Protoplasts purified from CaMV-infected leaves were placed on stretched Parafilm M membranes, covered with a cover glass and offered for acquisition feeding to aphids that had been starved for 1 hour. Figure 1 shows the experimental set-up. After a 1-hour acquisition period, aphids were transferred in groups of 10 insects to test plants for an inoculation period of 1 hour. Figure 2A shows that, under these conditions, on average ∼25% of the test plants were infected, although a rather large variation was observed. Shorter (15–45 min) acquisition times slightly but not significantly reduced transmission rates (*P* = 0.142), whereas longer acquisition times neither significantly increased nor reduced transmission. Thus, we concluded that a 60-min acquisition access period was a good compromise, which we now use as standard in transmission assays. We next tested the effect of pre-acquisition fasting on CaMV transmission. Aphids were starved for different time periods before they were allowed a 60-min acquisition feeding period on infected protoplasts. Figure 2B shows that fasting had only a marginal effect on transmission: aphids starved for only 15 to 45 min transmitted CaMV a little less efficiently than those starved for 60 min, but the difference was not significant (*P* = 0.083). On the other hand, prolonged starvation (90–240 min) did not increase transmission.

To examine more precisely the mechanism of virus uptake from infected cells, and the possible impact of the physiological state of the cell, it is essential that the aphids acquire virus particles directly from living cells and not from debris or contaminated medium. To check for this in our experimental set-up, protoplasts were disrupted by gentle ultrasonication (see Methods) before they were offered to aphids. Figure 2C shows that transmission was totally abolished under these conditions, suggesting that CaMV is not acquired from contaminated medium and that live cells are indeed required. As it might have been possible that ultrasonication inactivated viral genomes directly, the same sonicated protoplast suspension was employed in mechanical transmission assays. Figure 2D shows that ultrasonicated protoplasts were as infectious as control protoplasts in mechanical inoculation assays. This ruled out the possibility that the virus was inactivated by the treatment and further confirmed that aphids obligatorily had to acquire CaMV from living cells for transmission.

Because we previously showed that microtubules are required for formation of the TB [13], we tested the effect of oryzalin, a microtubule depolymeriser, on CaMV transmission. Most interestingly, this drug diminished transmission significantly (*P* = 0.014, Figure 2E). The effect of oryzalin was not due to increased protoplast mortality as measured by the fluorescein diacetate test [18] (data not shown). The effect of oryzalin was also not due to toxicity of the substance, because aphids transmitted CaMV after in vitro acquisition from a solution containing oryzalin and purified virus particles (as well as CaMV helper protein P2 and P3, required for transmission; see Figure 2F). Thus, we conclude that oryzalin exercises its effect on transmission by modifying the physiology of the cells, and is not an artefact.

**Figure 3:**
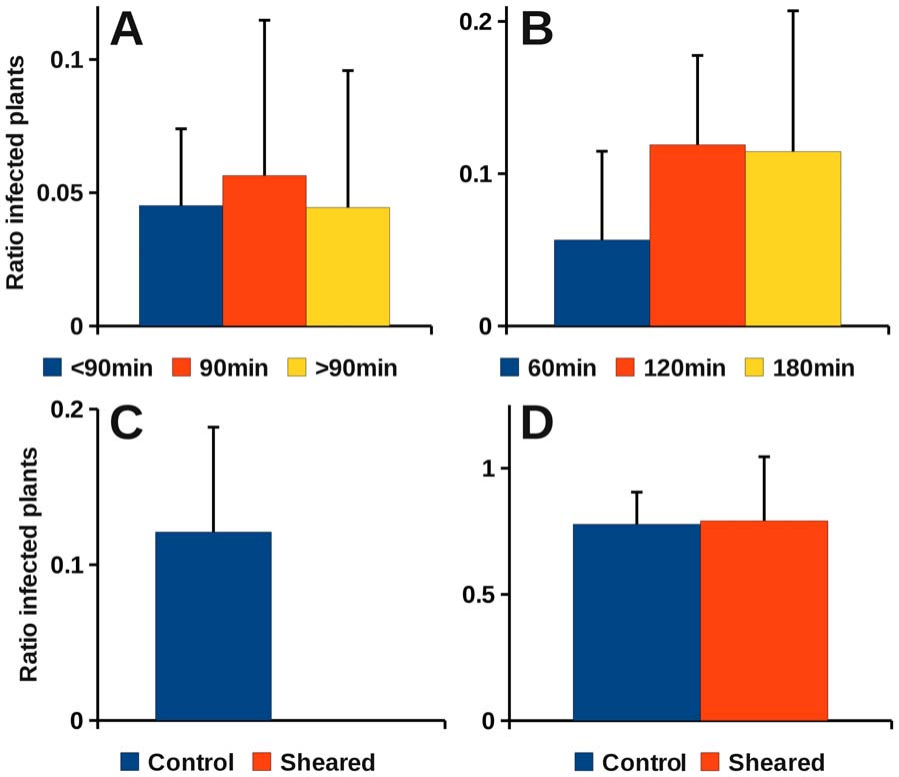
Protoplast transmission of TuMV. **A.** Effect of different acquisition times on TuMV transmission. After 60 min of starving, aphids were allowed different protoplast acquisition periods before being placed on test plants for inoculation. No effect of different acquisition time was observed (*P* = 0.942; df = 2; Kruskal-Wallis test). The graph combines data from 24 test plants per assay with 6 assays for acquisition times from 15 to 60 min (<90 min), 6 assays for an acquisition time of 90 min, and 4 assays for acquisition times between 120 and 180 min (>90 min). **B.** Pre-acquisition starving has no effect on TuMV transmission. Aphids were starved for 60, 120, or 180 min before being allowed a 60-min acquisition period on TuMV-infected protoplasts and subsequent transfer to test plants for inoculation. The graph shows that there is no measurable effect of pre-acquisition starving on transmission efficiency (*P* = 0.193; df = 2; Kruskal-Wallis test). 6 assays of 24 test plants were tested for 60 min starvation, 13 assays for the 120 min time point, and 4 assays for 180 min starvation. **C.** TuMV acquisition from protoplasts absolutely requires living protoplasts. Aphids were allowed to feed for 60 min on infected intact (control) or dead (sheared) protoplasts before transfer to test plants for inoculation. The difference between transmission rates from living and sheared protoplasts is highly significant (*P*<0.001; Mann-Whitney test). The graph shows data from 18 assays (9 for each condition) of 24 test plants each. **D.** Shearing does not inactivate TuMV. Infected protoplasts were homogenised by repeated passage through a syringe needle and then rub-inoculated to turnip test plants. The graph shows that virions from sheared protoplasts were as infectious as those from intact control protoplasts. Three 24 plant cultivation trays were inoculated per condition. All graphs present mean values ± standard deviation.

The protoplast-acquisition system might be of more general use if it also allows efficient transmission of viruses other than CaMV. So we tested transmission of a different non-circulative virus, turnip mosaic virus (TuMV)—one of the numerous species of the genus *Potyvirus*. Figure 3A shows that protoplasts infected with TuMV also allowed transmission by aphids, though transmission rates were lower than those obtained with CaMV. An acquisition time of 90 min seemed to be correlated with the highest transmission rate when compared to shorter or longer acquisition, but the differences were not significant (*P* = 0.942). We next tested the effect of aphid pre-acquisition starving on transmission of TuMV (Figure 3B). Like for CaMV, no major effect of starving was detected: after 120 min, transmission rates were slightly higher compared to 60 min pre-acquisition fasting but the difference was not significant (*P* = 0.193). Longer periods of starving did not increase transmission further. As in the case of CaMV, we wanted to prove that aphid acquisition of TuMV requires intact protoplasts. Because, in contrast to CaMV, ultrasound inactivates potyviruses, protoplast suspensions were passed repeatedly through a syringe needle, thus disrupting infected protoplasts by shearing. Disruption of cells was verified by wide field microscopy (not shown), and Figure 3C shows that it totally abolished aphid transmission. As with CaMV, we verified in mechanical rub inoculation assays that the disruption treatment did not inactivate TuMV particles (Figure 3D). Taken together, these results show that aphid transmission of TuMV from protoplasts also absolutely required living protoplasts.

## Discussion

Our results show unequivocally that living protoplasts can be used successfully as a source of virus in acquisition experiments, paving the way to pharmacological approaches to characterise vector-transmission. As a validation of this assay, we showed that disintegration of protoplasts totally abolished vector-transmission but not mechanical transmission of CaMV and TuMV. Further evidence for the biological relevance of the protoplast acquisition system comes from the observation that CaMV acquisition was significantly affected by depolymerisation of host cell microtubules with oryzalin. We have no definitive explanation for this phenomenon, but we can rule out that reduced transmission was caused by increased protoplast mortality or toxicity of oryzalin to aphids. We previously showed that the formation of intracellular CaMV TBs requires intact microtubules [13], but how microtubules actually relate to the very rapid acquisition process remains as yet unknown. In fact, it was this very question that prompted development of the assay described here, and our new experimental system will be useful for generating further results using other drugs and conditions in our quest to completely understand CaMV acquisition. In fact, the results of the simple experiment presented here illustrate that CaMV acquisition is not an incidental event where the cells serve as mere virus containers. Rather, some precise cellular physiological processes are obviously important at this exact moment. This is quite unexpected, particularly for non-circulative viruses, and our technique will be instrumental in investigating further the underlying phenomena not only in CaMV, but also in TuMV and perhaps other viruses. Infected protoplasts can be employed—after treatment with various bioactive compounds or drugs— in parallel in transmission assays and for phenotype analysis, for example, by fluorescence in situ hybridisation, immunofluorescence, and electron microscopy. This will allow us to confront and correlate the data obtained. This approach, for which we propose the name “virus-acquisition phenotyping assay” (VAPA), will allow us to connect host cell status directly to the efficiency of virus uptake and vector transmission. We want to point out, however, that protoplasts are somewhat different from intact cells in a tissue: they have for example no cell walls, different turgor conditions, and often altered gene expression patterns. This might in some cases impede transferability of findings from protoplasts to an intact organism.

Protoplasts have been used before in transmission experiments, for instance for whitefly-transmission of the non-circulative lettuce infectious yellows virus [19], and for aphid-transmission of the circulative turnip yellows virus [20]. In these two cases, protoplasts served only as a tool for generating virus particles; the protoplasts were disrupted to release virions into appropriate buffers compatible with subsequent acquisition by the vector. It is important to note that, in our case, disruption of protoplasts was carried out only directly into the culture medium, which is incompatible with in vitro acquisition of CaMV or TuMV. Actually, in vitro acquisition of CaMV and TuMV using purified virus particles and helper proteins or plant extracts requires specific buffers that maintain the helper proteins soluble [21], [22]. Our disrupted protoplasts would have supported transmission if we had used these specific buffers to resuspend the protoplasts. But by employing a protoplast culture medium, we established conditions in which virus acquisition exclusively from living cells could be monitored, thus allowing VAPA to be used to study virus acquisition under biologically relevant conditions. This is an entirely different and innovative objective of our system that distinguishes it from the two examples cited above.

Compared to more natural virus acquisition from infected plants, some differences were observed in VAPA that might intrigue readers familiar with non-circulative transmission. In plant-to-plant transmission of both CaMV and TuMV, the transmission rates reach a maximum after a few minutes of acquisition. Transmission rates do not change significantly for CaMV when acquisition time is increased, while they drop rapidly for TuMV after only 5–10 minutes [23]. While CaMV acquisition showed the expected pattern in our protoplast system, TuMV acquisition did not, because even after prolonged acquisition times (up to 3 hours) TuMV transmission did not drop. We believe that this discrepancy is caused by the structural differences between a protoplast suspension and an intact leaf. The former consists only of isolated cells in a liquid medium, in the latter the cells are organised into a three-dimensional tissue and are confined within cell walls. This doubtless results in different aphid feeding behaviour [24]. In the protoplast suspension, aphids aspire and ingest only from the homogeneous and uniform medium, and it is unknown whether and how they actually find cues to guide them towards living cells. In intact leaves aphids first insert their stylets into the median cell wall and then rapidly initiate numerous intracellular test probes, progressing gradually towards the sieve tubes. The protoplast system faithfully reproduces only one facet of the complex aphid feeding process—that of the intracellular stylet punctures. However, because many plant viruses are acquired or released during exactly this phase [2], VAPA should prove a valuable tool.

An unresolved question is how aphids are able to puncture with their stylets protoplasts that are floating freely in the medium. One would expect that they would push them away like a pool cue a ball, but successful virus acquisition shows that this is not the case. Possible explanations are that stylet movement is faster then the inertia of protoplasts in the medium, or that protoplasts are attracted to and stick by suction to the stylet tips during aspiration activity of the vector.

Another aspect that differs from previous reports on plant-to-plant transmission of TuMV is the effect of pre-acquisition fasting of aphids. While a 1–2 hour starvation period for the aphid vectors has no influence on subsequent plant-to-plant transmission of CaMV [25], which we confirm here, transmission of TuMV and of potyviruses in general is increased significantly under these conditions [26], [27]. Here, we were not able to observe an impact of pre-acquisition fasting of aphids on transmission of TuMV. Again we assume that the discrepancy is caused by the morphological differences between an intact leaf and the “cell-only” protoplast system. For example, it is easy to imagine that the longer period required by the aphid to find and puncture cells in the medium somewhat mimics pre-acquisition fasting. Alternatively, the amount of data collected was not sufficient to yield significant results for these experiments.

In conclusion, our protoplast system is reminiscent of one of the most important phases of the aphid-transmission process, i.e. intracellular puncture and the acquisition of viruses. It allows us to study acquisition from individual isolated cells exclusively, thus affording the possibility of discriminating between events taking place in cells and those occurring in cell walls and sieve tubes. Because, in contrast to entire leaves, protoplasts can be manipulated easily with different drugs, and their physiological state precisely controlled, the VAPA technique proposed here represents a powerful and totally novel tool with which to study virus acquisition on a cellular level, and will effectively allow the phenotyping of transmission. We demonstrate here that this tool can be applied efficiently to both caulimoviruses and potyviruses, and we predict that it could be transferred easily to other non-circulative plant viruses transmitted by aphids and other insects, and maybe even to circulative viruses, provided that they infect mesophyll cells, the principal source of protoplasts.
